# Producer knowledge and application of mineral supplementation in sheep farming systems

**DOI:** 10.3389/fvets.2025.1694107

**Published:** 2025-12-17

**Authors:** Alaina M. Helbus, Morgan S. Clemens, Samantha R. Yankocy, Claire Stenhouse

**Affiliations:** Department of Animal Science, Pennsylvania State University, University Park, PA, United States

**Keywords:** sheep, minerals, supplementation, reproduction, nutrition

## Abstract

**Introduction:**

Minerals are critical for ruminant health, reproduction, and productivity, yet deficiencies in grazing systems are common worldwide. This study characterized mineral supplementation practices among Pennsylvania sheep producers and evaluated their knowledge, perceptions, and diagnostic approaches.

**Methods:**

An anonymous Qualtrics survey, conducted from March to August 2024, yielded 168 valid responses from sheep producers statewide.

**Results:**

Most respondents managed small flocks (<20 ewes) and rated minerals as “very important” for both health (77.8%) and reproduction (78%). The majority (91.0%) provided supplemental minerals to their animals at certain or all stages of production. Commercial mineral mixes (72.0%) and salt/mineral blocks (29.8%) were the most frequently used delivery methods, with decisions primarily guided by producer experience, extension recommendations, and peer advice. Interestingly, only 30.7% of respondents had tested their soil/forage and only 9.0% had tested animals for mineral deficiencies, despite recognizing the importance of minerals for health and productivity. Reported health concerns included diarrhea, teeth grinding, and milk fever, while key reproductive challenges were mastitis, weak lambs, and stillbirths. Cost constraints, perceived adequacy of pasture, and flock size were cited as reasons for not supplementing.

**Discussion:**

These findings highlight strong awareness of mineral importance but limited adoption of diagnostic testing, suggesting potential gaps in targeted supplementation that may be more economical.

## Introduction

Minerals are essential for the regulation of numerous biological processes fundamental to animal health, growth, and development (1, 2). They are broadly categorized into macrominerals and microminerals (trace minerals). Macrominerals are required in relatively large quantities, typically expressed as grams per day or as a percentage of the diet, and fulfill critical structural, osmotic, and metabolic roles within the body. Examples of macrominerals include calcium, phosphorus, potassium, sodium, chlorine, magnesium, and sulfur. In contrast, microminerals are needed in much smaller amounts—generally expressed in milligrams or micrograms per day, or as parts per million in the diet—and function primarily as cofactors for enzymes and proteins. Key microminerals include iron, copper, zinc, manganese, iodine, selenium, and cobalt.

Globally, many ruminant production systems rely heavily on grazing, with livestock deriving most of their nutrients from pasture. Consequently, mineral deficiencies in soils and forages represent a widespread challenge with significant economic implications. The prevalence of mineral-deficient pastures varies according to region, soil characteristics, and plant species composition; however, inadequate mineral concentrations in grazing lands are consistently reported and have been shown to compromise livestock health and productivity (3). Deficiencies in cobalt, copper, selenium, iodine, phosphorus, and zinc .are common and are linked to reduced growth performance, impaired reproduction, compromised immune responses, and musculoskeletal abnormalities (4, 5).

Sheep production systems are often located on marginal lands and are subject to variable plant community composition, heterogeneous terrain, and challenging climatic conditions. Additionally, pastures in the U.S. have been extensively modified through the introduction of non-native forages aimed at improving yield or quality, the application of fertilizers, and management practices like overgrazing or hay production. These alterations have influenced mineral and vitamin availability to livestock, potentially leading to deficiencies or toxicities. These factors collectively influence the mineral content of forage and, consequently, the mineral supply to grazing animals (3). Thus, sustaining optimal productivity in grazing-based sheep systems is contingent upon ensuring that forage resources adequately meet the mineral requirements of the flock. Accurate assessment of mineral concentrations in soil, forage, and feed is therefore vital for formulating balanced rations, safeguarding animal health, and optimizing production efficiency.

Reproductive efficiency is a major determinant of profitability in sheep operations, with the number and weight of lambs weaned per ewe representing the primary driver of revenue. However, inefficiencies such as irregular estrous cyclicity, failure to establish pregnancy, embryonic or fetal loss, and compromised neonatal viability remain significant constraints on flock productivity. There is a well-established link between dietary mineral intake and reproductive performance in ruminants (6–17). Phosphorus deficiency, for instance, has been associated with delayed onset of puberty, irregular or absent estrous cycles, lower conception rates, reduced fertility, prolonged postpartum intervals, embryonic or fetal losses, and stillborn or weak offspring (6, 7, 11, 13–16). Conversely, phosphorus supplementation improves reproductive efficiency by promoting the resumption of estrous cycles and reducing the number of services required for conception in both cattle and sheep (16, 18, 19). Similar findings have been reported for other minerals, demonstrating a clear need to ensure producers understand the need for appropriate mineral intake in their operations, particularly during periods of high nutritional demands such as pregnancy and lactation. Nonetheless, producers must balance the need for adequate nutrient supply with the economic imperative of minimizing feed costs.

A variety of mineral supplementation strategies are available to sheep producers, examples of which include free-choice mineral blocks or loose mixes, in-feed fortification, water-medicated minerals, and injectable formulations. Each method presents distinct advantages and limitations in terms of intake regulation, cost-effectiveness, labor requirements, and ability to address specific deficiencies (3). The selection of an appropriate supplementation strategy depends on factors such as the type and severity of mineral deficiencies identified, flock size and management intensity, and prevailing environmental conditions. Importantly, there is evidence that many producers may oversupply minerals to ruminants (20, 21). Over-supplementation not only increases production costs but can also impair animal health and contribute to excess mineral excretion, with potential negative consequences for the environment.

The total United States sheep and lamb inventory was over 5 million head in January 2025, highlighting the importance of this industry for both nutrition and the economy in the United States (22). Pennsylvania consistently ranks in the top 15 states for sheep inventory, with producers marketing more than 60,000 lambs annually, contributing approximately $10 million to the state’s economy (22, 23). Despite the appreciation of the importance of nutrition for the success of the Pennsylvanian sheep industry, it is estimated that 90% of pastures used to raise sheep on in Pennsylvania fail to meet the nutrient benchmarks established by the National Research Council, being deficient in key minerals (24, 25). Consequently, supplementation is essential to meet the nutritional requirements of grazing sheep, particularly during physiologically demanding periods such as gestation and lactation. The present study aimed to characterize mineral supplementation practices among sheep producers in Pennsylvania and to assess their knowledge and perceptions regarding mineral deficiencies and supplementation approaches.

## Materials and methods

The institutional review board (IRB) at Pennsylvania State University deemed the study (STUDY00024065) exempt from formal IRB review.

### Survey distribution

A survey of sheep producers in Pennsylvania, USA was performed. Survey data was collected anonymously through Qualtrics (Qualtrics, Provo, UT, USA) during the period of March 1st, 2024, until August 31st, 2024. Individuals were recruited for this survey through email, social media, and in-person events. Producers were informed that the goal of this survey was to assess the use of mineral supplementation in breeding ewes in Pennsylvania. All producers had to confirm that they were over 18 years old and employed in sheep production in Pennsylvania. Additionally, we asked that each farm only submitted one survey. Responses that did not meet those criteria were excluded.

### Survey design

The survey questions asked to producers are described in Supplementary Table 1. These survey questions aim to gather information about sheep producers’ flock demographics, management practices, and perspectives on mineral supplementation. The survey questions explore producers’ knowledge, attitudes, and behaviors related to mineral use, particularly in relation to sheep health and reproduction, and seek to identify common reproductive challenges and the utilization of diagnostic practices such as soil or animal testing to inform mineral supplementation practices.

### Data management and statistical analysis

In total, 168 responses met the inclusion criteria for the study. The anonymized survey data were exported into an Excel spreadsheet (Microsoft Corporation, Redmond, WA.). The percentages reported in this paper are of those who responded to the question being reported (unless otherwise indicated). Descriptive statistics were generated, and data were visualized utilizing GraphPad Prism (Version 10, GraphPad, San Diego, CA).

## Results

### Flock demographics

There was a broad distribution of flock sizes among respondents (Table 1). A total of 26.8% of farms maintained 0–10 ewes, while 42.9% maintained 11–20 ewes. Flocks of 21–50 ewes were reported by 15.5% of respondents, and 6.6% managed 51–150 ewes. Only 8.3% of farms maintained more than 150 ewes.

**Table 1:** Flock demographics.

Descriptor	Percentage of respondents (%)
Flock size, no. of ewes	
0–10	26.8
11–20	42.9
21–50	15.5
51–150	6.6
151+	8.3
Number of lambs reared to market or as flock replacements per year	
0–10	27.9
11–20	19.1
21–50	23.8
51–150	25.0
151+	7.7
Farm type classification	
Sheep only	50.6
Sheep and goats	22.6
Sheep and beef	14.9
Other	11.9
Most common breeds	
Babydoll	6.6
Dorset	10.2
Suffolk	10.1
Cross breeds	23.8
Katahdin	25.6
Breed classification	
Meat only	47.9
Wool only	7.9
Dual-purpose only	21.8
Meat and wool	6.1
Meat and dual-purpose	12.1
Wool and dual-purpose	3.0
Wool, meat, and dual-purpose	1.2

The annual number of lambs reared to market or retained as flock replacements also varied considerably (Table 1). Nearly half (47.0%) of respondents reported rearing fewer than 20 lambs per year, including 27.9% with 0–10 lambs and 19.1% with 11–20 lambs. Medium outputs of 21–50 lambs were reported by 23.8% of respondents, and 25.00% produced 51–150 lambs annually. Consistent with the distribution of flock sizes, only 7.70% reported rearing more than 150 lambs per year, indicating that high-output flocks were uncommon in the surveyed population.

Respondents were asked to classify their operations based on the types of animals reared (Table 1). Overall, 50.6% reported producing sheep only, followed by sheep and goats (22.6%), sheep and beef cattle (14.9%), and other combinations (11.9%). Pennsylvania sheep producers reported utilizing a wide variety of breeds, including wool, meat, and dual-purpose types. The most frequently cited breeds were Babydoll Southdown, Dorset, Suffolk, Katahdin, and crossbreeds, with an additional 37 breeds reported (Supplementary Figure 1). Breed information was further categorized by production purpose (meat, wool, or dual-purpose) using classifications from the Oklahoma State University Extension.^1^ As shown in Table 1, most respondents raised either meat or dual-purpose breeds in their operations.

### Knowledge of mineral supplementation and importance for animal health and reproduction

**Figure 1:**
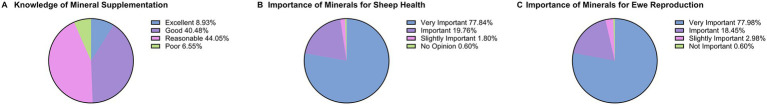
Respondent knowledge. **(A)** Respondents were asked to describe their knowledge of mineral supplementation. **(B)** Respondents were asked to state how important minerals are for animal health. **(C)** Respondents were asked to state how important minerals are for ewe reproduction.

Survey participants were asked to assess their knowledge of mineral supplementation (Figure 1A). The majority perceived themselves as having a moderate to strong understanding, with 44.1% rating their knowledge as “reasonable” and 40.5% as “good.” Smaller proportions of respondents reported “excellent” (8.9%) or “poor” (6.6%) knowledge.

Survey participants were asked to rate the importance of minerals for sheep health (Figure 1B). The majority (77.8%) considered minerals to be “very important,” and 19.8% rated them as “important.” Only 1.8% viewed minerals as “slightly important,” and 0.6% reported having no opinion. Similarly, survey participants were asked to rate the importance of minerals for ewe reproduction (Figure 1C). Most of the respondents (78%) considered minerals to be “very important,” while 18.5% rated them as “important” for ewe reproduction. Smaller proportions of respondents described minerals as “slightly important” (2.9%) or “not important” (0.6%). Collectively, these findings indicate a strong consensus among respondents regarding the critical role of minerals in supporting both animal health and ewe reproductive performance.

### Supplementation behaviors

**Figure 2:**
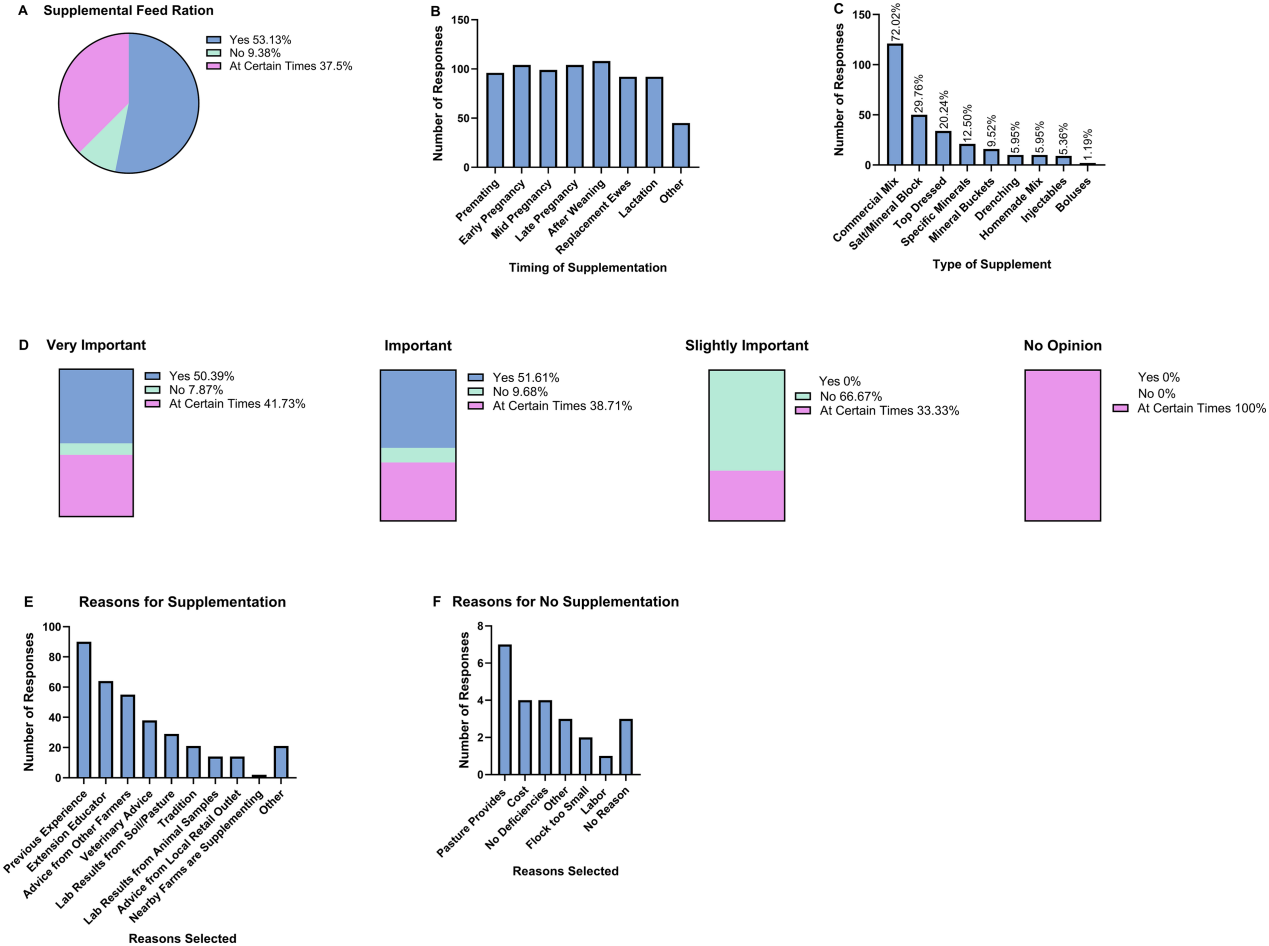
Survey responses regarding supplemental feed and mineral supplementation practices among ewe producers. **(A)** Distribution of respondents based on their provision of supplemental feed. **(B)** Timing of mineral supplementation across production stages. **(C)** Types of mineral supplementation used. **(D)** Relationship between perceived importance of mineral supplementation and its utilization. **(E)** Sources of information influencing mineral supplementation decisions. **(F)** Reasons for not providing mineral supplementation.

Respondents were surveyed regarding their provision of supplemental feed to their flocks (Figure 2). Half of the respondents (53.1%) reported consistently providing supplemental feed, while 37.5% indicated intermittent supplementation. A minority (9.4%) reported not providing any supplemental feed. These findings suggest that most respondents perceive pasture alone as insufficient to meet the nutritional requirements of their ewes.

A total of 91% of survey respondents provided mineral supplements to their ewes either continuously or during certain period of production. Survey participants were asked to select when they provide mineral supplementation to ewes (Figure 2B) and mineral supplementation was provided at high incidence throughout production in the survey population.

Respondents were asked to select all types of mineral supplementation they utilize (Figure 2C). Commercially available mineral mixes were selected by 72.0% of respondents. Other commonly utilized strategies included salt or mineral blocks (29.8%), top-dressed feed (20.2%), and specific mineral formulations (12.5%). Less frequently reported methods comprised mineral buckets (9.5%), drenching (6%), homemade mixes (6%), injectables (5.4%), and boluses (1.2%).

We then wanted to assess the relationship between how important the respondents believe mineral supplementation is for animal health and their utilization of mineral supplementation (Figure 2D). Among those who considered minerals to be very important, half reported consistent supplementation (50.4%), while 41.7% indicated use at certain times and 7.9% reported no use. Similarly, respondents who viewed minerals as important reported regular supplementation (51.6%), conditional use (38.7%), or no use (9.7%). In contrast, respondents who considered minerals to be only slightly important reported no regular supplementation (0%), with the majority indicating no use (66.7%) and a smaller proportion reporting use at certain times (33.3%). Respondents with no opinion did not report consistent or complete non-use, but all indicated conditional use (100%). These results suggest that perceived importance of minerals is closely associated with the likelihood of mineral supplementation.

Respondents who provide mineral supplementation to their ewes were asked to select the sources of information and advice that influenced their decision to supplement (Figure 2E). The most frequently reported source was previous experience (*n* = 90), followed by extension educators (*n* = 64), recommendations from other farmers (*n* = 55), and veterinary advice (*n* = 38). Laboratory analyses of soil or pasture samples (*n* = 29) and animal samples (*n* = 14) were less commonly utilized. Additional influences included tradition (*n* = 21), local retail outlets (*n* = 14), and practices observed on neighboring farms (*n* = 2). “Other” was selected by 21 respondents and examples of reasons provided included to reduce grain usage, improve known deficiencies, increase grain palatability, balance rations, improve wool and meat quality, improve reproductive success, and maintain body condition.

Among the 15 respondents who reported not providing supplemental minerals, the most commonly cited reason was reliance on pasture as an adequate nutrient source (*n* = 7; Figure 2F). Additional factors that were selected by respondents included cost constraints (*n* = 4), perceived absence of mineral deficiencies in their flock (*n* = 4), small flock size (*n* = 2), labor (*n* = 1), and “other” (*n* = 3). Of the 15 respondents, three indicated that they had no reason for not providing mineral supplementation.

### Reproductive and health challenges that may be associated with mineral deficiencies

**Figure 3:**
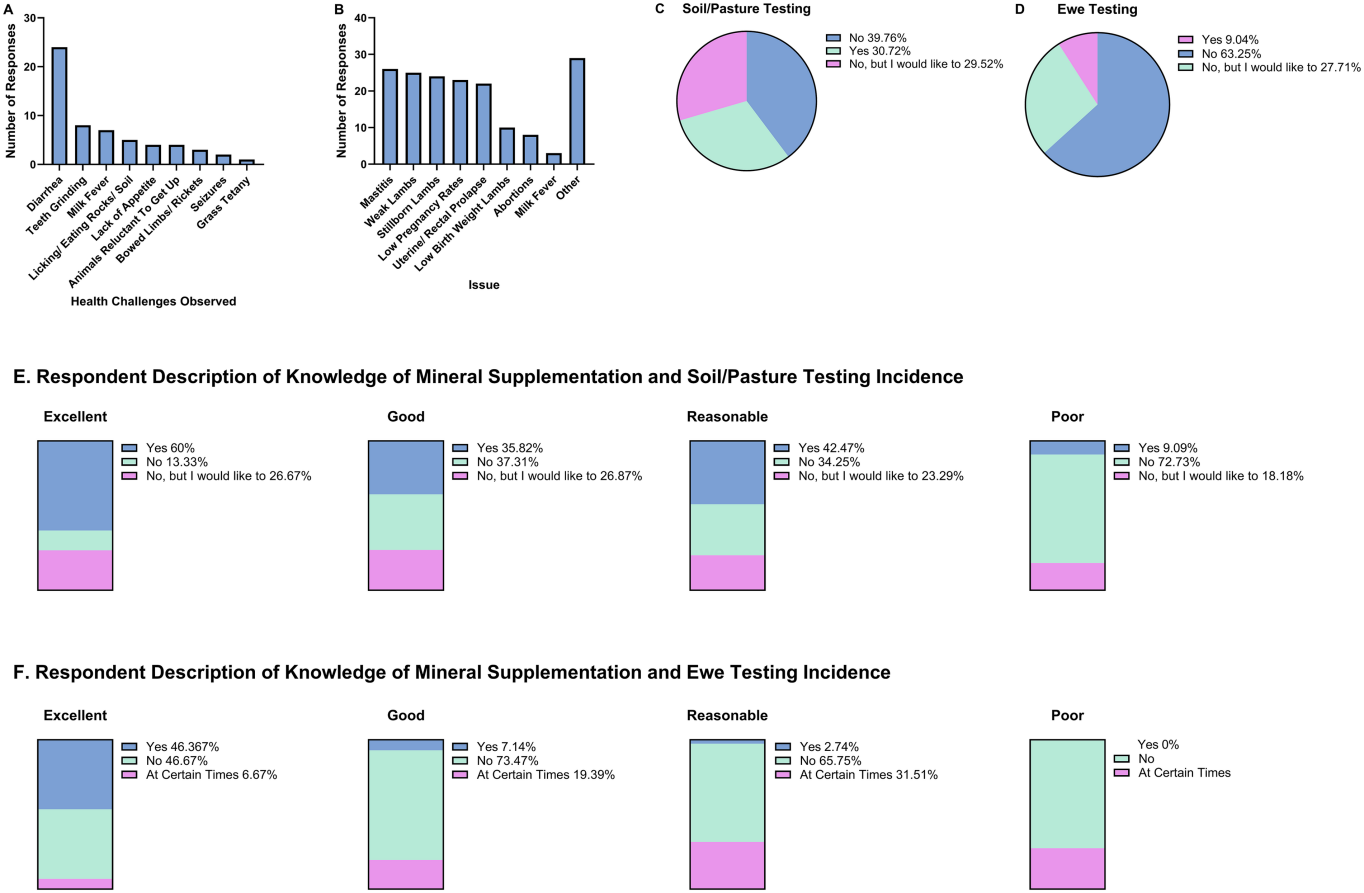
Survey responses regarding health challenges, reproductive issues, and mineral testing practices among ewe producers. **(A)** Frequency of observed health conditions potentially indicative of mineral deficiencies. **(B)** Top reproductive challenges reported by respondents. **(C)** Soil and pasture mineral testing practices. **(D)** Ewe mineral deficiency testing practices. **(E)** Relationship between self-reported knowledge of mineral supplementation and soil/pasture testing. **(F)** Relationship between self-reported knowledge of mineral supplementation and ewe testing.

As minerals are essential for many processes, mineral deficiencies can have severe consequences for animal health. A list of symptoms potentially indicative of mineral imbalances or deficiencies in sheep was provided to survey participants and they were asked to select all that they routinely observe in their operations (Figure 3A). No respondents selected convulsions, muscle tremors, spine abnormalities, paralysis, head pressing, depression, weakness/lethargy or aimless walking. Overall, the prevalence of any of the conditions listed among the respondents was low. Diarrhea was the most frequently observed condition (*n* = 24), followed by teeth grinding (*n* = 8), milk fever (*n* = 7), and abnormal ingestion behaviors, including licking or consuming rocks or soil (*n* = 5). Additional reported signs comprised lack of appetite (*n* = 4), reluctance to rise (*n* = 4), bowed limbs or rickets (*n* = 3), seizures (*n* = 2), and grass tetany (*n* = 1).

There is a well-established link between nutrition, particularly mineral intake, and reproductive outcomes. Survey participants were asked to indicate the top three reproductive challenges they experience (Figure 3B). The most frequently reported concerns included mastitis (*n* = 26), weak lambs (*n* = 25), stillbirths (*n* = 24), and low pregnancy rates (*n* = 23). Uterine or rectal prolapse was noted by 22 respondents, while fewer producers reported low birth weight lambs (*n* = 10), abortions (*n* = 8), and milk fever (*n* = 3) as important reproductive issues in their flock. Importantly, “Other” was selected by 29 respondents. Under the “Other” category of reproductive issues, respondents reported a diverse array of concerns, including low lambing rates per ewe, maternal neglect, poor body condition scores, dystocia and breach lambs, breed-specific challenges, timing of breeding, oversized lambs complicating parturition, milk fever, insufficient milk production, post-weaning mastitis, stillbirths associated with triplet births, and suspected viral etiologies such as Cache Valley Virus.

The mineral content of soil and pasture plays a critical role in determining the mineral status of grazing ruminants. Therefore, assessing soil/pasture mineral levels is a key strategy to ensure that ruminants receive adequate amounts of essential minerals to meet their nutritional requirements. Survey participants were asked whether they had conducted testing of their soil or pasture mineral composition (Figure 3C). Of the respondents, 39.8% indicated that they had not tested their soil or pasture, while 30.7% reported having conducted soil or pasture testing. An additional 29.5% expressed interest in testing in the future.

Similarly, survey participants were asked if they had tested their ewes for mineral deficiencies (Figure 3D). Most respondents (63.3%) reported not having tested their ewes for mineral deficiencies, while 27.7% indicated an interest in conducting such testing in the future. Only 9.0% of respondents reported having performed mineral deficiency testing.

We then wanted to assess the relationship between how respondents would describe their level of knowledge of mineral supplementation and whether they test their soil/pasture for mineral deficiencies (Figure 3E). For soil and pasture testing, 60% of respondents who described their knowledge as “excellent” reported testing, 13.33% did not test, and 26.7% expressed interest in testing. Among those with “good” knowledge, 35.8% reported testing, 37.3% did not, and 26.9% indicated interest. Respondents who rated their knowledge as “reasonable” reported testing at a similar rate (42.5%), while 34.3% did not test and 23.3% expressed interest. In contrast, among those who considered their knowledge “poor,” only 9.1% reported testing, 72.7% did not, and 18.2% expressed interest in testing.

Similarly, we wanted to assess the relationship between how respondents would describe their level of knowledge of mineral supplementation and whether they test or would be interested in ewes for mineral deficiencies (Figure 3F). For ewe testing, respondents with “excellent” knowledge reported the highest testing rates, with 46.4% conducting tests, 46.7% not testing, and 6.7% testing at certain times. Those who described their knowledge as “good” reported lower ewe testing, with 7.1% testing, 19.49% testing at certain times, and 73.5% not testing. Among those with “reasonable” knowledge, only 2.7% reported ewe testing, while 31.5% tested at certain times and 65.8% did not test. Respondents with “poor” knowledge did not report any ewe testing, though 27.3% indicated testing at certain times and the remainder reported no testing. These results indicate that higher self-reported knowledge of mineral importance is associated with more frequent soil/pasture and ewe testing, while limited knowledge corresponds with reduced or absent testing practices.

## Discussion

Mineral nutrition is a cornerstone of ruminant health, reproduction, and productivity, yet deficiencies remain a common challenge in grazing systems worldwide. Although mineral supplementation is widely used to mitigate the risk of clinical deficiencies, the specific practices, delivery methods, and producer knowledge underlying supplementation strategies are not well characterized. While most producers recognized the importance of minerals and routinely provided supplementation, reliance on generalized practices and anecdotal guidance was more common than evidence-based diagnostic testing. The low adoption of soil, forage, and animal testing suggests that supplementation programs may not always align with flock-specific needs, raising the risk of inefficiencies in production practices. These findings underscore a gap between producer awareness and the implementation of targeted strategies, highlighting opportunities for improved education efforts to support more precise and cost-effective mineral management.

A wide variety of sheep breeds were represented in this survey, encompassing meat, wool, and dual-purpose types. The current National Research Council (NRC) requirements for sheep (26) does not differentiate mineral requirements among these categories, yet it is plausible that physiological differences could influence nutritional needs. Furthermore, many operations reported managing multiple livestock species. Mixed-species operations add complexity to on-farm mineral supplementation strategies as ruminant species differ markedly in both mineral requirements and tolerance (26–28). Consequently, mineral programs formulated for one species may be inadequate—or even harmful—for another. For example, sheep are particularly sensitive to copper, selenium, and iodine toxicity (26). This challenge is particularly relevant in small-scale operations, where multiple species are often housed and fed together, underscoring the need for tailored nutritional management strategies.

Respondents demonstrated strong awareness of the role of minerals, with nearly 80% rating supplementation as “very important” for both health and reproduction. Most self-rated their knowledge as “reasonable” or “good,” suggesting an informed producer base. This apparent disconnect between perceived knowledge and evidence-based practice suggests that education efforts should emphasize how testing can reveal hidden or subclinical deficiencies that may impact productivity.

Almost all producers (91%) reported providing mineral supplementation either consistently or at specific production stages. This aligns with recommendations for continuous or targeted supplementation to support key physiological periods such as gestation and lactation. Commercial mineral mixes were by far the most common method (72%), followed by salt/mineral blocks and top-dressing. More labor-intensive or customized delivery methods—such as drenching, injectables, and boluses—were rare, possibly reflecting time constraints, ease-of-use priorities, or cost considerations.

Decisions about supplementation were driven primarily by previous experience, extension advice, and peer recommendations, with laboratory-based data playing a secondary role. This reliance on experiential knowledge may be effective in preventing overt deficiencies—as suggested by the low prevalence of classic deficiency signs in the survey population—but may also contribute to unnecessary supplementation.

Although respondents demonstrated a strong appreciation for the importance of minerals in animal health, and the majority reported providing mineral supplementation to their ewes, only 30.7% had conducted soil or pasture testing, and just 9.0% had performed mineral deficiency testing on their ewes. These findings indicate a limited uptake of diagnostic mineral assessments, despite widespread recognition of the role of minerals in flock health and reproductive performance. Notably, a substantial proportion of respondents expressed interest in testing either the mineral composition of their soil/pasture or the mineral status of their ewes. This suggests that certain perceived barriers—such as cost, accessibility, or uncertainty in interpreting results—may hinder adoption of such practices. Further research should aim to identify and address these barriers, thereby enabling small ruminant producers to make more targeted and informed decisions regarding mineral supplementation strategies. The lack of testing raises the possibility that some producers may be over-supplementing minerals. Excessive mineral provision increases feed costs, can negatively affect animal health, and contributes to environmental concerns through elevated excretion of minerals such as phosphorus and copper (1, 3, 21). Given the high rate of supplementation across the production cycle in this population of respondents, even modest adjustments based on diagnostic results could reduce costs and environmental impact without compromising animal performance.

These findings underscore the importance of education programs in closing the gap between producers’ general awareness of mineral nutrition and their adoption of diagnostic tools. Educational initiatives should directly address common misconceptions about pasture sufficiency by emphasizing the risk of subclinical deficiencies, even on well-managed land. They should also highlight the economic benefits of diagnostic testing, demonstrating how precision supplementation can reduce unnecessary expenditures, while offering practical guidance on sampling procedures, interpretation of results, and application in management decisions. In addition, providing species- and breed-specific recommendations through education and extension will be particularly valuable for mixed-species operations. Future research should focus on quantifying the prevalence of subclinical mineral imbalances in Pennsylvania flocks and exploring their associations with reproductive performance and lamb growth rates. Establishing clear links between mineral testing outcomes and production metrics may offer compelling evidence to encourage more widespread adoption of tailored supplementation strategies.

## Conclusion

Sheep producers demonstrate strong commitment to mineral supplementation, primarily via commercially available mixes, and high awareness of its importance for flock health and reproduction. However, the low uptake of diagnostic testing limits the precision of supplementation programs and may result in both under- and over-supply of key minerals. Education efforts that promote cost-effective testing and provide breed- and production-type specific guidance could enhance both economic and environmental sustainability while supporting optimal animal performance.

## Data Availability

The original contributions presented in the study are included in the article/Supplementary material, further inquiries can be directed to the corresponding author.

## Footnotes

1
https://breeds.okstate.edu/sheep/

## Supplementary material

SUPPLEMENTARY FIGURE 1 Summary of least common breeds reported by respondents.

## References

[1] López-Alonso M. Trace minerals and livestock: not too much not too little. ISRN Vet Sci. (2012) 2012:1–18. doi: 10.5402/2012/704825, 23762589 PMC3671743

[2] Wu G. Principles of animal nutrition. Boca Raton, FL: CRC Press (2018).

[3] Stewart WC Scasta JD Taylor JB Murphy TW Julian AAM. Invited review: mineral nutrition considerations for extensive sheep production systems. Appl Anim Sci. (2021) 37:256–72. doi: 10.15232/aas.2021-02143

[4] Merck & Co. Inc. Merck veterinary manual. 11th ed. Kenilworth, NJ: Merck & Co. Inc. (2016).

[5] Pugh D. G. (2020). Nutritional requirements of sheep: Minerals and vitamins Columbus, Ohio: The Ohio State University, Small Ruminant Team. Available online at: https://u.osu.edu/sheep/2020/07/28/nutritional-requirements-of-sheep-minerals-and-vitamins/

[6] Call JW Butcher JE Supe JL Blake JT Olson AE. Dietary phosphorus for beef cows. Am J Vet Res. (1986) 47:475–81. 3954238

[7] Eckles CH Gullickson TW Palmer LS. Phosphorus deficiency in the rations of cattle. Minneapolis, Minnesota: University of Minnesota Agricultural Experiment Station (1932).

[8] Gonzalez-Rivas PA Lean GR Chambers M Liu J. A trace mineral injection before joining and lambing increases marking percentages and lamb weights on diverse farms in Victoria, Australia. Animals. (2023) 13:178. doi: 10.3390/ani13010178, 36611786 PMC9817843

[9] Harvey KM Cooke RF Marques R d S. Supplementing trace minerals to beef cows during gestation to enhance productive and health responses of the offspring. Animals. (2021) 11:1159. doi: 10.3390/ani11041159, 33919507 PMC8072782

[10] Hidiroglou M. Trace element deficiencies and fertility in ruminants: a review. J Dairy Sci. (1979) 62:1195–206. doi: 10.3168/jds.S0022-0302(79)83400-1, 387829

[11] Hignett SL Hignett PG. The influence of nutrition on reproductive efficiency in cattle. Part II. The effect of the phosphorus intake on ovarian activity and fertility of heifers. Veterinariya. (1952) 84:203–6.

[12] Morrow DA. Phosphorus deficiency and infertility in dairy heifers. J Am Vet Med Assoc. (1969) 154:761–8. 5812997

[13] O’Moore L. Aphosphorosis in Ireland. Nature. (1950) 165:192.

[14] Phiri ECJH Nkya R Pereka AE Mgasa MN Larsen T. The effects of calcium, phosphorus and zinc supplementation on reproductive performance of crossbred dairy cows in Tanzania. Trop Anim Health Prod. (2007) 39:317–23. doi: 10.1007/s11250-007-9016-2, 17944301

[15] Read MVP Engels EAN Smith WA. Phosphorus and the grazing ruminant. 1. The effect of supplementary P on cattle at Glen and Armoedsvlake. S Afr J Anim Sci. (1986) 16:1–6.

[16] Steevens BJ Bush LJ Stout JD Williams EI. Effects of varying amounts of calcium and phosphorus in rations for dairy cows. J Dairy Sci. (1971) 54:655–61. doi: 10.3168/jds.S0022-0302(71)85902-7, 5105953

[17] Van Emon M Sanford C McCoski S. Impacts of bovine trace mineral supplementation on maternal and offspring production and health. Animals. (2020) 10:12. doi: 10.3390/ani10122404, 33339123 PMC7765511

[18] Senosy W Kassab AY Hamdon HA Mohammed AA. Influence of organic phosphorus on reproductive performance and metabolic profiles of anoestrous Farafra ewes in subtropics at the end of breeding season. Reprod Domest Anim. (2018) 53:904–13. doi: 10.1111/rda.13183, 29733477

[19] Taylor B. Selke M. Swingle S. Phillips J. Vinson H. 1976 Phosphorus levels for beef cows during lactation and breeding., in Proceedings from Arizona cattle feeder’s day

[20] Kendall NR Holmes-Pavord HR Bone PA Ander EL Young SD. Liver copper concentrations in cull cattle in the UK: are cattle being copper loaded? Vet Rec. (2015) 177:493. doi: 10.1136/vr.103078, 26489996 PMC4680191

[21] Sinclair LA Atkins NE. Intake of selected minerals on commercial dairy herds in central and northern England in comparison with requirements. J Agric Sci. (2015) 153:743–52. doi: 10.1017/S0021859614001026

[22] USDA (United States Department of Agriculture National Agricultural Statistics Service). 2025. Available online at: https://www.nass.usda.gov/.

[23] Barkley M. E. Hartman D. W. Kime L. F. Harper J. K. (2023). Off Season and Accelerated Lamb Production. Available online at: https://extension.psu.edu/off-season-and-accelerated-lamb-production (Accessed August 23, 2023).

[24] Fournier M. 2023. Feeding the Flock. Available online at: https://extension.psu.edu/feeding-the-flock (Accessed August 23, 2023).

[25] Muller L. D. 2023. Dietary minerals for cows on pastures. Available online at: https://extension.psu.edu/dietary-minerals-for-dairy-cows-on-pasture (Accessed August 23, 2023).

[26] NRC. Nutrient requirements of small ruminants: Sheep, goats, cervids, and new world camelids. Washington, DC: The National Academies Press (2007).

[27] NRC. Nutrient requirements of beef cattle. Eighth ed. Washington, DC: The National Academies Press (2016).

[28] NRC. Nutrient requirements of dairy cattle. Washington, DC: National Academy Press (2021).

