# Supplementary figures and images for: Producer knowledge and application of mineral supplementation in sheep farming systems
Source: Front Vet Sci. 2025 Dec 17;12:1694107. doi: 10.3389/fvets.2025.1694107 (PMC12753353; doi:10.3389/fvets.2025.1694107)

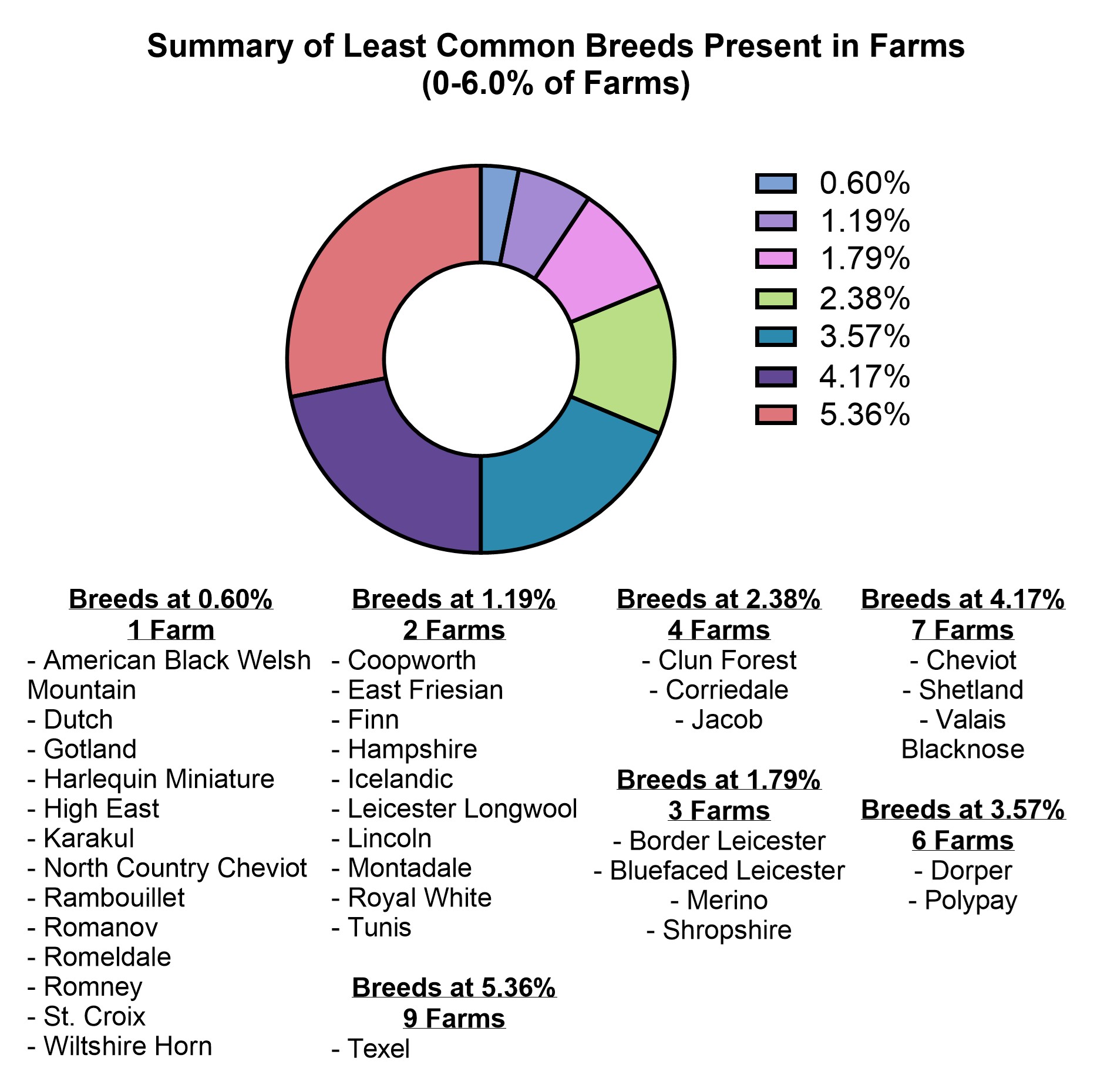

Supplement: SUPPLEMENTARY FIGURE 1 — Summary of least common breeds reported by respondents. [file Image_1.jpg]
