# Supplementary material for: Producer knowledge and application of mineral supplementation in sheep farming systems
Source: Front Vet Sci. 2025 Dec 17;12:1694107. doi: 10.3389/fvets.2025.1694107 (PMC12753353; doi:10.3389/fvets.2025.1694107)
Supplement: Supplementary file 2 [file Table_1.docx]

**Supplementary Table 1: Summary of Questions Asked in the Survey**

| **Questions Asked** | **Options Available to Respondents** |
| --- | --- |
| How many ewes do you have? | Short answer response |
| What breeds of sheep do you have? | Short answer response |
| How many lambs do you rear to market weight or as flock replacements per year? | Short answer response |
| How would you classify your operation? | Sheep only, Sheep and Goats, Sheep and Beef, Sheep and Dairy, Other (write in) |
| In addition to pasture, do you provide a supplemental feed ration (e.g. concentrate feed) to your flock? | Yes, No, At Certain Times (Write in more information) |
| How would you describe your knowledge of mineral supplementation? | None, Poor, Reasonable, Good, Excellent |
| How important do you think minerals are for sheep health? | Not Important, Slightly Important, Important, Very Important, No opinion |
| How important do you think minerals are for ewe reproduction? | Not Important, Slightly Important, Important, Very Important, No opinion |
| Do you provide mineral supplements to your animals? | Select all that apply.  None, Salt/mineral block, Mineral buckets, Commercial mineral mix, Specific minerals, Homemade mineral mix, Drenching, Extra minerals mixed into feed or top dressed, Boluses, Injectables, Other (write in) |
| If you do not provide mineral supplementation, what is the reason? Select all that apply. | Select all that apply.  Pasture and/or feed provides adequate minerals, No known mineral deficiencies in flock, Flock too small, Flock too large, Cost, Labor, No reason, Other (write in) |
| If you provide mineral supplements, what are the reasons behind your decision to provide mineral supplements? Select all that apply. | Select all that apply.  Veterinary advice, Extension educator/other agricultural professional advice, Advice from local retail outlet (merchant, co-op, supply store, feed mill), Advice from other farmers, Tradition, Laboratory results of soil/pasture, Laboratory results from samples from your animals, Nearby farms are supplementing minerals, Previous experiences (opportunity to provide more written information), Other (write in) |
| When do you give your ewes mineral supplements? Select all that apply. | Select all that apply.  Never, Premating, Early Pregnancy, Mid Pregnancy, Late Pregnancy, Lactation, After weaning, Replacement Ewes, Other (write in) |
| What are the main reproductive challenges you regularly experience? | Indicate top 3.  Low Pregnancy Rates, Abortions, Stillborn Lambs, Low Birth Weights, Weak Lambs, Milk Fever, Mastitis, Uterine/Rectal Prolapses, Other (write in) |
| Do you routinely observe any of the following in your flock? | Milk Fever, Grass tetany, Seizures, Convulsions, Muscle tremors, Bowed limbs, Rickets, Swayback/Spine abnormalities, Paralysis, Head pressing, Curved/bowed spines, Depression, Diarrhea, Weakness/Lethargy, Teeth grinding, Lack of appetite, Animals not wanting to get up, Licking/eating rocks and soil, Aimless walking |
| Have you tested your soil/pasture mineral composition? | Yes, No, No but I would like to |
| Have you tested your ewes for mineral deficiencies? | Yes, No, No but I would like to |
